# Combining Reduced Irrigation with Organic Fertilizer Substitution Enhances Water–Nitrogen Productivity and Soil Carbon–Nitrogen Pools of Maize in Arid Northwest China

**DOI:** 10.3390/plants15152270

**Published:** 2026-07-24

**Authors:** Wei Pan, Fuqiang Li, Xiaofan Pan, Wenbo He, Weijie Shi, Jianlong Wei, Qinli Wang, Haoliang Deng

**Affiliations:** 1 Gansu Provincial Engineering Research Center for the Resource Utilization of Edible Fungi and Fungi Bran, Hexi University, Zhangye 734000, China; panwei2025666@163.com (W.P.); panxiaofan2023@163.com (X.P.); wangqinli66@163.com (Q.W.); 2 College of Water Conservancy and Hydropower Engineering, Gansu Agricultural University, Lanzhou 730070, China; lifuq@gsau.edu.cn (F.L.); hewenbo2025@163.com (W.H.); 3 College of Agriculture and Ecological Engineering, Hexi University, Zhangye 734000, China; 4 Agricultural Technology Extension Center of Ganzhou District, Zhangye 734000, China; shiweijie202688@163.com (W.S.); weijianlong2026@163.com (J.W.)

**Keywords:** maize, deficit irrigation, organic fertilizer substitution, water–nitrogen productivity, soil carbon pool

## Abstract

To address the challenges of excessive water and fertilizer application, declining soil fertility, and suboptimal maize yields in the oasis irrigation areas of Northwest China, a two-year (2024–2025) field experiment was conducted. Three irrigation levels were implemented: a 30% reduction (W1: 3575 m^3^·ha^−1^), a 15% reduction (W2: 4335 m^3^·ha^−1^), and conventional irrigation (W3: 5100 m^3^·ha^−1^). Nitrogen management comprised three strategies: 30% organic substitution (N3), 15% organic substitution (N2), and full chemical nitrogen fertilizer (N1). Statistical approaches, including correlation analysis and the CRITIC-AHP-VIKOR comprehensive evaluation model, were applied. The results indicated that ear length differed significantly among treatments, with the longest ears observed in W2N2 and W3N2 (17.39–17.68 cm) and the shortest in W1N1 (14.52 cm). Under W2 conditions, the kernel number per ear in N2 was 1.73% higher than that in N3, whereas under W1 conditions, N2 showed a 0.21% reduction compared to N3. The 100-kernel weight varied minimally, ranging from 32.04 to 40.70 g, with no significant difference between W2N2 and W3N3. Among all treatments, W2N2 produced the greatest yield, exceeding those of W3N3 and W2N3 by 6.07% and 5.96%, respectively. During the two experiments years, this treatment also promoted the accumulation of soil carbon and nitrogen in the 0–20 cm layer, increasing soil organic matter by 22.34–118.15% and total nitrogen by 5.62–40.45%. In contrast, grain quality parameters, including crude protein, crude starch, and crude fat contents, did not differ significantly between W2N2 and W3N3. Moreover, W2N2 achieved an irrigation water use efficiency of 4.41 kg·m^−3^, which was significantly greater than that under W1N2, while nitrogen use efficiency was improved by 42.57%. Correlation analysis revealed that maize yield is significantly positively correlated with soil carbon and nitrogen pools, grain quality indicators, and nitrogen fertilizer partial productivity, whereas quality parameters were positively correlated with irrigation water use efficiency, though not significantly. The CRITIC-AHP-VIKOR model identified that the comprehensive strategy of 15% irrigation reduction combined with 15% organic nitrogen substitution synergistically enhances maize yield, maintains soil carbon and nitrogen pools, and improves water–nitrogen use efficiency. These results provide a theoretical basis for promoting sustainable maize cultivation in arid irrigated areas characterized by limited water and nitrogen availability.

## 1. Introduction

Maize (*Zea mays* L.) serves as a globally important crop that plays a significant role in food production, animal feed, and industrial processing, and is indispensable to maintaining the stability of the global food supply [1,2]. According to data from the Food and Agriculture Organization of the United Nations (FAO), annual global maize production has surpassed 1.2 billion tons. China is a major maize-producing country, with both its planting area and total output ranking prominently among cereal crops worldwide [3]. Domestically, maize stands as the major grain crop in terms of both cultivation area and total yield. In 2024, the maize cultivation area in China had expanded to approximately 4.47 × 10^7^ ha, with a yield of 2.95 × 10^8^ tons, accounting for 45.20% of the total grain yield [4]. However, in arid and semi-arid regions, especially the Hexi Oasis irrigation areas, which are important maize production bases in China, the annual average rainfall is less than 200 mm, and agricultural production is highly dependent on irrigation. The traditional flood irrigation method not only leads to severe waste of water resources, but also results in an irrigation water use efficiency of only 0.8–1.2 kg·m^−3^, which is far lower than the level of 2.0 kg·m^−3^ in developed countries [5,6]. At the same time, in pursuit of high yields, excessive fertilization is widespread (more than 300 kg·ha^−1^). This results in a nitrogen fertilizer use rate of only 30–35%, with large amounts of nitrogen being lost through ammonia volatilization, nitrification–denitrification and leaching. Such resource losses can result in a range of environmental problems, particularly soil quality degradation and eutrophication of aquatic ecosystems [7,8,9]. Previous studies have demonstrated that increasing the nitrogen application rates beyond 200 kg·ha^−1^ does not produce a significant further increase in maize yield, but instead causes a marked reduction in the agronomic efficiency of applied nitrogen fertilizer [10]. Therefore, improving the coordinated and efficient use of water and nitrogen while maintaining high maize productivity has become a key scientific challenge for promoting green agriculture development and achieving high-yield and resource-efficient grain production in arid and semi-arid regions.

In response to the above issues, scholars, both domestic and foreign, have conducted extensive research on irrigation regulation and fertilizer optimization. Studies have confirmed that deficit irrigation can induce the physiological adaptation mechanism of crops under moderate water stress conditions, thereby improving water use efficiency without significantly reducing yield. Previous studies have demonstrated that moderate water deficit (maintaining soil moisture at 60–70% of the field capacity) imposed during the vegetative growth stage of maize could improve crop physiological performance compared with full irrigation. Under such conditions, chlorophyll concentration and leaf area index increased by 9.1–22.2% and 5.1–19.3%, respectively, accompanied by 2.6–6.3% enhancement in final grain yield [11,12,13]. Furthermore, Li et al. [14] revealed that the interaction between water and nitrogen supply substantially regulates the dry matter partitioning and grain yield formation of maize. At a nitrogen application rate of 200 kg·ha^−1^, maize yield reached 1336 kg·ha^−1^, relative to this treatment, reducing nitrogen input to 100 kg·ha^−1^ increased yield by 16.1%, whereas increasing nitrogen input to 300 kg·ha^−1^ resulted in a 9.2% yield improvement. In response to soil degradation caused by the long-term excessive application of chemical nitrogen fertilizers (CNF), Li et al. [15] conducted a meta-analysis across 48 field trial sites. The results indicated that simply reducing the application rate of CNF led to a 3.83% decrease in soil organic matter (SOM) and an 11.46% decrease in total nitrogen (TN). In contrast, partially replacing CNF with organic materials has become a viable strategy for improving soil fertility and crop productivity [16,17]. For instance, studies conducted in semi-arid producing regions showed that substituting organic fertilizer (OF) for 30% of the CNF input increased grain yield and nitrogen use efficiency by 9.2% and 27.8%, respectively [18]. Long-term field trials on the Loess Plateau further indicated that the integrated application of organic and mineral fertilizers enhanced SOM content in the surface layer by 15–41%, accompanied by a corresponding increase in TN [19]. Regarding grain quality, Niewegłowski et al. [20] reported that increasing the nitrogen application rate from 0 to 160 kg·ha^−1^ raised the crude protein concentration of maize kernels by 18.96 g·kg^−1^. Similarly, research on pumpkins production in the arid regions of northwestern China confirmed interactions between irrigation and fertilization exert significant effects on crop quality and fertilizer use efficiency [21].

Previous research has investigated the effects of irrigation regulation and OF substitution on crop growth, generally treating these two practices independently. However, most studies have primarily emphasized crop yield responses. SOM and TN are key indicators of soil fertility and long-term productive capacity; however, their dynamics under integrated water and fertilizer management have not yet been fully elucidated. Meanwhile, a systematic understanding is still lacking in how reduced irrigation and organic fertilizer substitution jointly affect grain quality, irrigation water use efficiency (IUE), and nitrogen partial factor productivity (NFP), as well as whether synergistic or trade-off relationships exist among these objectives. To alleviate excessive irrigation water consumption, overapplication of CNF, low nitrogen use efficiency, and declining soil quality in the arid agricultural regions of northwestern China, this study develops an integrated water–fertilizer management strategy combining deficit irrigation with partial OF substitution. The independent and interactive effects of these management practices on the soil–crop system are systematically investigated. The study aims to achieve the goals of water-saving, nitrogen reduction, and efficiency improvement, with a particular focus on the water–fertilizer synergy effect under the conditions of reduced irrigation, CNF reduction, and OF improvement in soil micro-environment conditions. This study systematically evaluated the combined effects of the reduced irrigation and OF substitution on the soil–crop system, with particular emphasis on: (1) variations in SOM and TN contents; (2) maize yield and its components, including ear length, kernel number per ear, and 100-kernel weight; (3) the formation of major grain quality attributes, namely crude protein, crude starch, and crude fat content; and (4) the synergistic improvement mechanism of IUE and NFP. Finally, through a comprehensive evaluation model, the optimal combination of irrigation amount and the application rates of CNF and OF was selected, which takes into account high-yield, high-quality, and efficient resource use. The findings provide a technical basis for sustainable agricultural water management and the mitigation of agricultural non-point source pollution in the arid areas of northwestern China.

## 2. Results

### 2.1. Effects of Reduced Irrigation and OF Substitution on SOM and TN

#### 2.1.1. Soil Organic Matter

**Figure 1:**
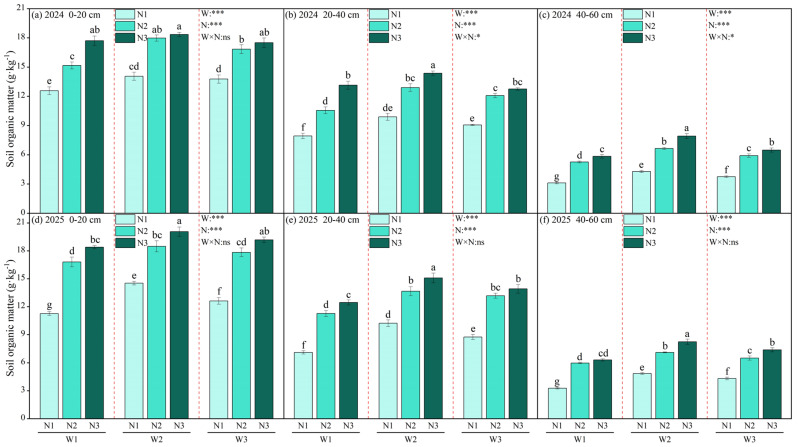
Effects of reduced irrigation and OF substitution on SOM in 0–60 cm soil layer of maize. Note: (**a**–**c**) show SOM in the 0–20, 20–40, and 40–60 cm layers, respectively, in 2024; (**d**–**f**) present the corresponding soil layers in 2025. Bars represent mean values, and error bars indicate standard errors (*n* = 3). Different lowercase letters denote significant differences among the treatments at *p* < 0.05. * and *** indicate significant differences among the different treatments at the levels of *p* < 0.05, and *p* < 0.001, respectively. ns means not significant at the level of *p* ≥ 0.05.

Under the synergistic effect of reduced irrigation and OF substitution for CNF, there were changes in SOM in the 0–60 cm soil layer (Figure 1). Overall, SOM content significantly decreased with increasing soil layer depth, reaching the highest level in the topsoil layer (0–20 cm) and the lowest in the subsoil layer (40–60 cm). This distribution pattern indicates that the accumulation of SOM is mainly confined to the plow layer (0–20 cm). The two-year data indicate that both the irrigation water volume and the proportion of OF replacing CNF have significant effects on the SOM content in the 0–60 cm layer of the maize field (*p* < 0.001), but the interaction between the two does not reach a significant level (*p* > 0.05). Across all soil layers, the SOM content in 2025 was generally higher than that in 2024, suggesting that the continuous implementation of this agronomic practice of replacing CNF with organic materials can gradually increase SOM content, particularly under the optimal coupling application mode. By comparing the differences in SOM among different treatments resulting from reduced irrigation and OF substitution for CNF, it can be seen that under the same irrigation amount, increasing the application rate of OF significantly increases SOM content, and this trend is most prominent at the W2 level. In the topsoil layer, the W2N3 treatment exhibited the highest SOM content, reaching 20.07 g·kg^−1^ in 2025, and there was significant difference compared with the W2N1 and W2N2 treatments. This indicates that under the W2 level, the carbon enrichment effects of N3 were significantly superior to the low OF treatment. At the W1 and W3 levels, as the amount of OF applied increased, the increase in SOM content in the low irrigation water treatment was greater than that in the high irrigation water treatment. Moreover, the SOM content of W3N3 did not significantly exceed that of W2N2. A comparison among different irrigation amounts indicates that, under the same OF application rate, the W2 level generally outperformed the W1 and W3 levels. Taking the N2 level as an example, in the topsoil layer, the SOM content of W2N2 was significantly higher than that of W1N2, and was comparable to that of W3N2.

#### 2.1.2. Total Nitrogen

**Figure 2:**
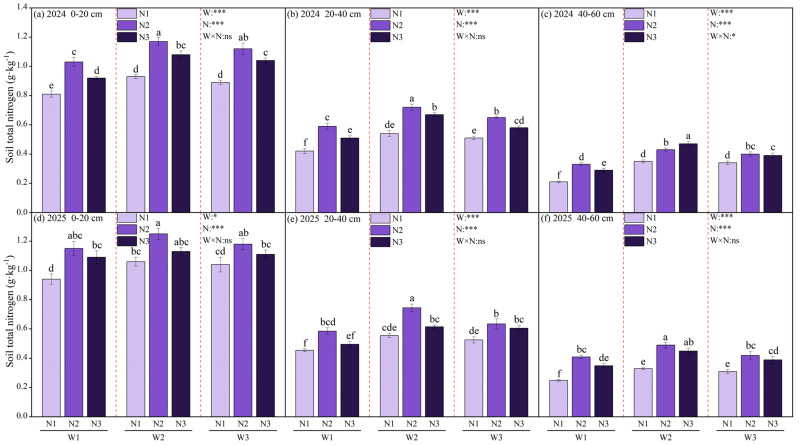
Effect of reduced irrigation and OF substitution on TN content in the 0–60 cm soil layer. Note: (**a**–**c**) show TN content in the 0–20, 20–40, and 40–60 cm soil layers, respectively, in 2024; whereas panels (**d**–**f**) present the corresponding soil layers in 2025. Bars represent mean values, and error bars indicate standard errors (*n* = 3). Different lowercase letters denote significant differences among the treatments at *p* < 0.05. * and *** indicate significant differences among the different treatments at the levels of *p* < 0.05, and *p* < 0.001, respectively. ns means not significant at the level of *p* ≥ 0.05.

The effect of reduced irrigation combined with OF substitution for CNF on soil TN content in the 0–60 cm soil layer is shown in Figure 2. Soil TN content exhibits significant spatial aggregation characteristics, with the highest content in the topsoil layer, and is decreasing with increasing soil layer depth. The two-year result indicated that irrigation amount and OF substitution each exerted a highly significant effect on TN content throughout the 0–60 cm soil profile (*p* < 0.001). However, no significant interaction between these two factors was detected for TN content at any depth within this profile (*p* > 0.05). In 2025, the total nitrogen content across all treatments was generally higher than that in 2024, indicating a significant cumulative effect of nutrient management. In the topsoil layer (0–20 cm), the combination of moderate reduced irrigation (W2) with medium-to-high rates of OF substitution for CNF (N2 and N3) showed the most prominent effects in the efficient utilization of nitrogen. Among these, W2N2 achieved a TN content of 1.25 g·kg^−1^ in 2025, demonstrating the optimal synergistic effect alongside the W2N3 and W3N2 treatments. It is worth noting that at the W2 level, increasing the organic fertilizer application rate from N2 to N3 did not result in a significant increase in TN content, indicating that there is a reasonable application threshold. In contrast, the treatment with W3 combined with N1 resulted in a significantly lower TN content, highlighting the fundamental role of organic inputs in maintaining the soil nitrogen pool.

### 2.2. Effects of Reduced Irrigation and OF Substitution for CNF on Maize Yield and Its Components

The effects of reduced irrigation combined with OF substitution for nitrogen fertilizer on maize yield and its components are shown in Table 1. During the two years from 2024 to 2025, irrigation amount had a highly significant effects (*p* < 0.001) on yield and 100-kernel weight. The irrigation amount also has a highly significant impact on the ear length and the kernel number per ear (*p* < 0.01). The proportion of OF replacing CNF had only a significant effect (*p* < 0.05) on the ear length, had a highly significant effect (*p* < 0.01) on the 100-kernel weight, but had no significant effect on the kernel number per ear and yield (*p* > 0.05). The interaction between irrigation volume and the replacement ratio had no significant effect on maize yield and its constituent factors (*p* > 0.05). In terms of yield components, both W1N3 and W1N1 were lower than W3N3. The average ear length of W2N2 over two years was 17.68 cm, which was comparable to the average ear length of 17.11 cm for W3N3. The average kernel number per ear of W2N2 was 528 grains, which was 4.35% higher than that of W3N3 (506 grains). The average 100-kernel weight of W2N2 was 40.70 g, which was similar to that of W3N2 (40.38 g). The effects of reduced irrigation combined with organic fertilizer substituting CNF on yield components varied depending on the irrigation amount, nitrogen application rate, and OF substitution rate. At the W1 level, increasing the OF substitution rate had only a limited effect on kernel number per ear. The average kernel number per ear of W1N3 was 469 grains, which was only 0.21% higher than that of W1N2 (468 grains). At the W2 level, the kernel number per ear of W2N2 was 1.73% higher than that of W2N3 (519 grains). The final yield analysis showed that the two-year average yield of W2N2 was 19.56 t·ha^−1^, which was higher than that of W3N3 (18.44 t·ha^−1^), with an increase of 6.07%. Meanwhile, it was 5.96% higher than that of W2N3 (18.46 t·ha^−1^), a high OF substitution treatment under the same irrigation level. It is worth noting that under the W1 level, the average yield of W1N3 (13.53 t·ha^−1^) was significantly lower than that of W2N3.

### 2.3. Effects of Reduced Irrigation and OF Substitution for CNF on Grain Quality

**Figure 3:**
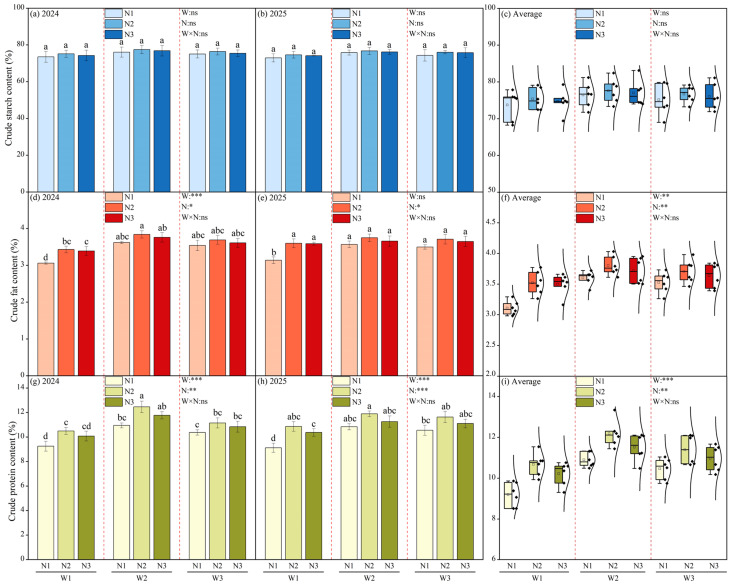
Effects of reduced irrigation and OF substitution for CNF on grain quality. (**a**,**b**) respectively represent the impact of reducing irrigation and substituting OF for CNF on the crude starch content in 2024 and 2025; (**c**) indicates the impact of reducing irrigation and substituting OF for CNF on the average crude starch content over the two years. (**d**,**e**) respectively represent the impact of reducing irrigation and substituting OF for CNF on the crude fat content in 2024 and 2025; (**f**) indicates the impact of reducing irrigation and replacing CNF with OF on the average crude fat content over the two years. (**g**,**h**) respectively represent the impact of reducing irrigation and substituting OF for CNF on the crude protein content in 2024 and 2025; (**i**) indicates the impact of reducing irrigation and replacing OF with CNF on the average crude protein content over the two years. Different lowercase letters denote significant differences among the treatments at *p* < 0.05. *, **, and *** indicate significant differences among the different treatments at the levels of *p* < 0.05, *p* < 0.01, and *p* < 0.001, respectively. ns means not significant at the level of *p* ≥ 0.05.

The effects of reduced irrigation and OF substituting CNF on maize quality are shown in Figure 3. The results indicated that different treatments had varying impacts on maize grain quality parameters. In terms of crude starch content, both reduced irrigation and OF substituting CNF did not show significant effects (*p* > 0.05), and their interaction effect did not reach a significant level. Overall, crude starch content showed an increasing trend with the increase in the amount of OF and irrigation. The two-year average value of crude starch content was 75.61% at the W3N3 and 77.11% at the W2N2, which was 1.98% higher than that of W3N3. At the W1 level, the effect of crude starch content reduction was more obvious. The average crude starch content at the W1N1 (73.25%) was 1.90% lower than that of W3N1 (74.67%). Regarding crude fat content, the effects of OF substituting nitrogen fertilizer were significant (*p* < 0.05), but their interaction effect was not significant (*p* > 0.05). Crude fat content increased with the increase in the amount of OF and irrigation. The two-year average crude fat content of W3N3 was 3.64%. It is worth noting that, among the CNF reduction treatments, the average crude fat content at the W2N2 was 3.80%, which was 4.40% higher than that of W3N3. During the maturity stage of 2024–2025, the crude protein content of maize was significantly influenced by the amount of OF substituting CNF and irrigation. The two-year data show that W2N2 achieved the maximum value of crude protein (with an average of 12.18%), followed by W2N3, which increased by 6.89% compared to W3N2, and 4.97% compared to W3N3. Analysis of variance indicated that crude protein content was significantly influenced by the level of OF substituting CNF and irrigation amount. Under constant irrigation levels, as the proportion of OF substitution increases, the crude protein content first rises and then decreases, but the decrease is much smaller than the increase. It is worth noting that the average crude protein content of the N3 was 7.07% higher than that of N1 and 4.47% lower than that of N2. Among each organic fertilizer level, the crude protein content increased first and then decreased with increasing irrigation amount, declining after reaching a peak. The optimal irrigation amount was W2, whose crude protein content was 14.96% and 5.39% higher than that of W1 and W3, respectively.

### 2.4. Effects of Reduced Irrigation and OF Substitution for CNF on IUE and NFP of Maize

**Figure 4:**
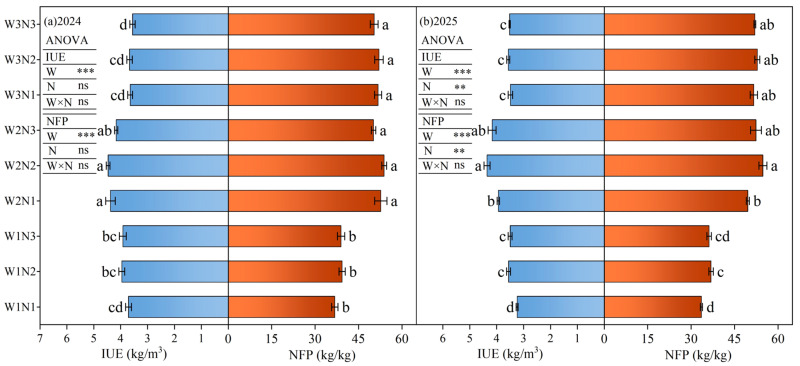
Effects of reduced irrigation and OF substitution for CNF on IUE and NFP of maize. Note: (**a**) represents the effects of reduced irrigation and OF substitution for CNF on IUE and NFP of maize in 2024; (**b**) represents the effects of reduced irrigation and OF substitution for CNF on IUE and NFP of maize in 2025. Different lowercase letters in the same column indicate significant differences among the different treatments (*p* < 0.05). **, and *** indicate significant differences among the different treatments at the levels of *p* < 0.01, and *p* < 0.001, respectively. ns means not significant at the level of *p* ≥ 0.05.

The effects of reduced irrigation and OF substitution for CNF on maize IUE and NFP are shown in Figure 4. Reduced irrigation had highly significant effects on IUE (*p* < 0.001). The effect of OF substitution on IUE and NFP only reached a significant level (*p* < 0.05) in 2025. The interaction effect between reduced irrigation and OF substitution did not reach a significant level (*p* > 0.05) in terms of IUE and NFP. The IUE of reduced irrigation treatments (W1 and W2) was generally higher than that of W3 treatment. Based on the two-year average, the IUE of W2N2 was the highest, reaching 4.41 kg·m^−3^, which was significantly higher than that of W3N3 (3.54 kg·m^−3^), representing an increase of 24.82%. In terms of nitrogen fertilizer use, under the same reduced irrigation treatments, the NFP for the N2 treatments was consistently and significantly higher than those for the N1 treatment, with no significant difference between N3 and N2. At the W2 level, the two-year average NFP of W2N2 was 54.32 kg·kg^−1^, which was 5.93% higher than that of W2N3, with the two-year average NFP of W2N3 was 51.28 kg·kg^−1^. Analysis of the interaction effect between irrigation amount and organic fertilizer and other nitrogen substitution rates indicated that, at the W1 level, the IUE of W1N3 (3.71 kg·m^−3^) was not significantly different from that of W1N2 (3.76 kg·m^−3^). Its NFP (37.58 kg·kg^−1^) was also lower than that of the N2 treatments under the same irrigation amount.

### 2.5. Correlation Analysis

**Figure 5:**
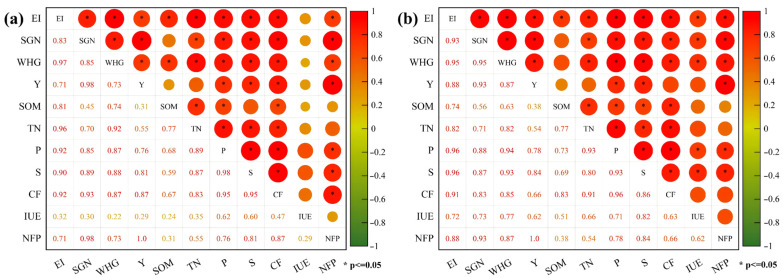
Correlation analysis of maize water–nitrogen productivity and soil carbon–nitrogen pool indicators under reduced irrigation and OF substituting CNF. (**a**) represents the correlation of maize water–-nitrogen productivity and soil carbon–-nitrogen pool indicators in 2024; (**b**) represents the correlation of maize water–-nitrogen productivity and soil carbon–-nitrogen pool indicators in 2025. *EI* represents ear length; *SGN* represents kernel number per ear; *WHG* represents 100-kernel weight; *Y* represents yield; *SOM* represents soil organic matter; *TN* represents total nitrogen; *P* represents crude protein; *S* represents crude starch; *CF* represents crude fat; *IUE* represents irrigation water use efficiency; *NFP* represents nitrogen fertilizer partial productivity. * indicates significant differences among the different treatments at the levels of *p* < 0.05.

The correlation analysis of maize yield indicators, soil carbon and nitrogen pool indicators, quality, and water–nitrogen use efficiency under the coupling of reduced irrigation and organic fertilizer substituting nitrogen fertilizer is shown in Figure 5. The correlation analysis was conducted using the mean values for each treatment. Across the two maize growing seasons, ear length was significantly and positively correlated with kernel number per ear, 100-kernel weight, grain yield, SOM, TN, and grain crude protein content, crude starch content, and crude fat content (*p* < 0.05). Kernel number per ear also showed significantly positive relationships with 100-kernel weight, yield, TN, and all three grain quality parameters, indicating that greater kernel set was associated with improved yield formation, soil nitrogen status, and grain quality. Similarly, 100-kernel weight was positively correlated with yield, SOM, TN, crude protein content, crude starch content, and crude fat content, suggesting a close association among grain filling, soil fertility, and quality formation. Grain yield was significantly and positively related to crude protein content and crude starch content. In the surface soil layer, SOM was significantly correlated with TN as well as grain crude protein content and crude fat content, implying that greater SOM accumulation may enhance soil nitrogen availability and contribute to improved maize nutritional quality. Soil TN was likewise significantly associated with all three grain quality parameters, indicating that improved soil nitrogen levels status was accompanied by enhanced grain quality. In addition, significantly positive correlations were observed among all three grain quality parameters, demonstrating coordinated variation among the principal and processing-quality attributes of maize grain. The crude starch content was significantly positively correlated with crude fat content, indicating that as crude starch accumulates, crude fat levels also increase correspondingly. Maize ear length, kernel number per ear, 100-kernel weight, yield, SOM, TN, crude protein content, crude starch content, and crude fat content were positively correlated with IUE, but the correlations were not significant (*p* > 0.05), indicating that an increase in irrigation efficiency is beneficial to some extent for improving yield components, soil fertility indicators and quality indicators. Maize output and its constituent factors, all three grain quality parameters were significantly positively correlated with NFP, indicating that an increase in NFP has a certain positive promoting effect on yield components, soil fertility, and grain quality. IUE was positively correlated with NFP, but the correlation does not reach a significant level, suggesting that there may be a certain trade-off between water efficiency and nitrogen efficiency, but this mutual constraint is not yet significant.

### 2.6. Comprehensive Evaluation: Optimization of Reduced Irrigation and Nitrogen Substitution Schemes Based on Organic Fertilizer

**Figure 6:**
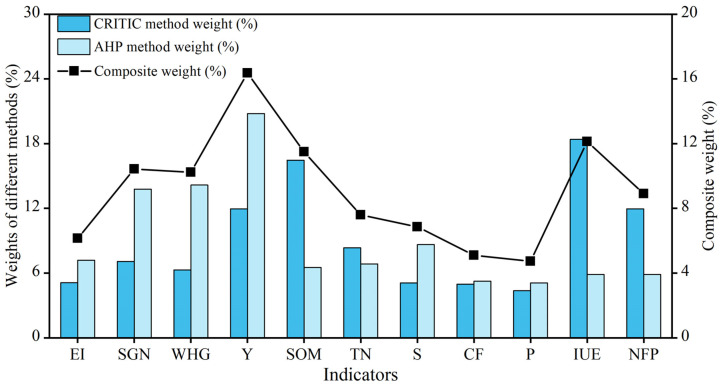
Weights of each indicator. Note: the indicator weights were calculated based on the average of data from 2024 to 2025.

Objective weight distribution has a positive effect on the credibility of the research results. However, from the perspective of actual agricultural production in this study, yield and quality should be the primary considerations. Therefore, this study adopts a combined approach of objective weighting and subjective weighting, and uses the CRITIC method and AHP method to determine the comprehensive weights of each indicator (Figure 6), and then employs the comprehensive evaluation method for multi-objective optimization. The CRITIC weight analysis method was used to calculate the weight of each indicator. The weights of ear length, kernel number per ear, 100-kernel weight, yield, soil organic matter, total nitrogen, crude starch content, crude fat, and NFP are 5.10%, 7.09%, 6.29%, 11.95%, 16.45%, 8.33%, 5.08%, 4.96%, and 11.95%, respectively. Among all indicators, IUE had the highest weight, accounting for 18.39%, while crude protein content had the lowest weight, accounting for 4.38%. The AHP method was used to analyze and obtain the weights of ear length, kernel number per ear, 100-kernel weight, yield, soil organic matter, total nitrogen, crude starch, crude fat, crude protein, IUE, and NFP, which are 7.19%, 13.79%, 14.16%, 20.78%, 6.52%, 6.84%, 8.64%, 5.25%, 5.08%, 5.87% and 5.88%, respectively. By weighting the weights obtained by the CRITIC method and the AHP method in a 1:1 ratio, the weights for ear length, kernel number per ear, 100-grain weight, yield, soil organic matter, total soil nitrogen, crude starch, crude fat, crude protein, IUE and NFP are 6.14%, 10.44%, 10.23%, 16.37%, 11.49%, 7.59%, 6.86%, 5.11%, 4.73%, 12.13%, and 8.91%, respectively. The comprehensive evaluation results based on the CRITIC-AHP-VIKOR model are shown in Table 2. The W2N2 treatment group exhibited the best performance in terms of group utility value (S = 0.015), individual regret value (R = 0.015), and benefit ratio value (Q = 0), ranking first overall in the comprehensive evaluation. W2N3 ranked second, with a S value of 0.148, an R value of 0.032, and a Q value of 0.126. W1N1 and W1N3 ranked lower, as the 9th and 8th respectively, with S values of 1.00 and 0.671, and R values of 0.164 and 0.143, being significantly higher than those of the optimal treatment. Among all reduced irrigation and OF substituting CNF treatments, the W2N2 treatment ranked first and had the best comprehensive performance.

## 3. Discussion

### 3.1. Effects of Reduced Irrigation and Organic Fertilizer Substitution on Soil Carbon and Nitrogen Pools

The combination of reduced irrigation and partial substitution of OF for CNF significantly increased SOM and TN content. Under the condition of reducing irrigation amount by 15% compared with conventional irrigation combined with the full amount of CNF, the treatment with 15% OF substitution significantly increased SOM and TN contents in the topsoil layer. This result is highly consistent with existing studies. Zhang et al. [22] conducted a six-year maize field experiment and found that, compared with conventional fertilization, the combination of OF application and reduced irrigation significantly increased SOM by 14.9% and total nitrogen by 106.7%, confirming the synergistic effect of water–fertilizer coupling on enhancing soil carbon and nitrogen pools. A seven-year study in a jujube orchard in the arid region of Northwest China showed that drip irrigation combined with OF significantly promoted the annual accumulation of SOM, with both its content and reserves being higher than those under conventional irrigation combined with CNF. Path analysis indicated that the input of OF was the most critical factor driving soil organic carbon accumulation (path coefficient was 0.65) [23]. In this study, reduced irrigation did not have a negative impact on SOM accumulation. Instead, it achieved an increase in SOM when combined with OF application. The reason may be that moderate water deficit reduced the mineralization rate of SOM, thereby decreasing carbon loss, while the continuous input of OF provided a stable carbon source for SOM accumulation [24]. In terms of the perspective of total nitrogen change, the treatment of OF substituting CNF significantly increased soil TN content. This is related to the fact that nitrogen in OF exists in an organic form and is slowly released through mineralization, thereby reducing nitrogen loss from leaching and volatilization [25]. Wang et al. [26] also confirmed in a semi-arid maize study that the combined application of OF and CNF significantly improved soil nitrogen supply capacity and promoted post-anthesis nitrogen uptake and translocation. In summary, reducing irrigation amount by 15% and substituting 15% of chemical nitrogen fertilizer with OF can achieve a synergistic regulation of carbon and nitrogen inputs. This approach maintains and enhances soil carbon and nitrogen pools whilst conserving water, representing an effective approach for water conservation and soil carbon–nitrogen pool enhancement in the Northwest irrigation district.

### 3.2. Effects of Reduced Irrigation and OF Substitution on Maize Grain Quality and Water–Nitrogen Productivity

The results of this study indicate that the coupling effect of reduced irrigation and OF substituting CNF has a significant regulatory effect on maize yield, grain quality, IUE, and NFP, with a clear interaction between the two factors. Compared with the combination of conventional irrigation and full CNF application, reducing irrigation amount by 15% combined with substituting 15% of CNF with OF can stabilize yield while significantly improving grain quality, as well as enhancing IUE and NFP. The advantages of this water–fertilizer combination may arise from the synchronization between appropriate soil moisture availability and the release of organic nutrients. A similar study showed that reducing irrigation by 20% and substituting 25% of mineral nitrogen with OF increased maize yield and biomass by 13.0% and 8.9%, respectively. Moderate irrigation promotes root nitrogen uptake and residue-derived carbon inputs, while organic fertilizer directly supplies organic carbon and nitrogen and improves soil water-holding capacity, thereby facilitating simultaneous increases in maize yield and soil carbon and nitrogen pools [27]. Furthermore, this study also found that replacing a portion of CNF with OF significantly increased the crude protein, crude starch, and crude fat contents of maize grains, which is consistent with the findings of earlier studies. For example, Li et al. [23] conducted a seven-year long-term experiment in the arid region of Xinjiang and found that the combination of drip irrigation and OF significantly increased the soluble solids and vitamin C contents of jujube, indicating that OF has unique advantages in improving the internal quality of fruits. Similarly, studies on greenhouse tomatoes showed that the combination of soluble OF and CNF can effectively reduce fruit nitrate content and enhance taste quality [28]. In this study, the improvement in grain quality under OF substitution may be associated with the slow and sustained release of nutrients from OF, which could better match maize nutrient demand during the late grain-filling stage, potentially prolonging leaf functional duration and promoting the translocation of photosynthetic assimilates to the grains. However, these physiological processes were not directly measured and therefore require further verification [29].

Based on the findings regarding IUE and NFP, the reduced irrigation treatments in this study did not lead to a decrease in water productivity. Instead, under conditions of appropriate OF application, water productivity was actually improved. As with studies on maize, moderate deficit irrigation can induce stomatal regulation in crops, reduce excessive transpiration, and thereby improve water use efficiency [26]. In this experiment, the treatment with 15% reduction in irrigation amount had significantly higher irrigation water productivity than conventional irrigation, which is consistent with the findings of Zhang et al. [30] in their study on silage maize. It is worth noting that the improvements in IUE and NFP in this study were not attributable to a single factor, but rather resulted from the synergistic optimization of water and fertilizer. A study on the coupling of organic fertilizer and reduced irrigation for maize in the arid region of Northeast China showed that the coupling effect of OF and reduced irrigation can promote post-anthesis nitrogen uptake and translocation in maize and increase the nitrogen harvest index [26]. Studies on sunflower in saline–alkali soils have also confirmed that drip irrigation combined with organic fertilizer can significantly reduce nitrate nitrogen leaching while improving NFP [31]. In this study, it is speculated that the synergistic effect of deficit irrigation and OF substitution may improve soil aggregate structure and enhance soil water and nutrient retention capacity, thereby promoting efficient absorption and the use of water and nutrients by plants [23]. It is worth noting that although the TN input for each treatment in this study was the same, the available nitrogen supply in the current season might be different. Organic nitrogen needs to be mineralized by microorganisms before it can be absorbed by crops, and its release may not be synchronized with the peak nitrogen demand of maize in the early stage. Under condition W1, water shortage may also inhibit organic nitrogen mineralization, root growth, and nitrogen transport. Therefore, the yield reduction in W1N3 might be caused by both water stress and insufficient available nitrogen in the current season. Although a higher proportion of organic fertilizer substitution is beneficial for total nitrogen accumulation in the soil and long-term soil improvement, it may temporarily reduce the supply of mineral nitrogen in the current season. Since the dynamic of mineral nitrogen and the net mineralization rate were not measured in this study, this mechanism still needs to be further verified.

Furthermore, the analysis identified a critical threshold in the substitution ratio of OF. Beyond this threshold, the effect on yield and quality changes significantly. The comprehensive performance of the 15% substitution ratio was superior to that of the 30% substitution ratio, which aligns with the findings of Shen et al. [32] on winter wheat. An excessively high substitution ratio of organic fertilizer may lead to insufficient supply of available nutrients during the early stages of crop growth, affecting the development of vegetative organs, thereby restricting yield potential in the later stages. The study by Chen et al. [33] on tomatoes also showed that the combined application of OF and CNF could improve fruit quality without reducing yield. However, applying OF alone can enhance the quality of the fruits, but it often leads to a decrease in yield. In summary, the coupling model of reducing irrigation amount by 15%, combined with substituting 15% of CNF with OF through the synergistic regulation of water and fertilizer, can maintain stable maize yield while improving grain quality, and enhance IUE and NFP. This represents a viable approach to conserving resources and improving quality in irrigation areas of Northwest China.

### 3.3. Optimization of Water-Saving and Nitrogen-Reducing Organic Fertilizer Management for Maize

This study employed a multi-objective optimization comprehensive evaluation framework based on the CRITIC-AHP-VIKOR model to systematically assess the effects of deficit irrigation and OF substituting CNF on soil carbon and nitrogen pools in the 0–60 cm soil layer, yield and its components, grain quality, and water and nitrogen use efficiency under drip irrigation conditions. The results showed that moderately reducing irrigation amount combined with organic fertilizer application could maintain crop yield while significantly enhancing soil carbon and nitrogen pools, thereby improving grain quality, IUE, and NFP. These results are consistent with those reported by Ma et al. [34] and Xiao et al. [7]. The comprehensive evaluation model employed in this study can effectively integrate indicators such as yield, soil carbon and nitrogen pools, quality, and water and nitrogen use efficiency, thereby providing quantitative decision support for nitrogen reduction and organic fertilizer application management. This is consistent with the evaluation methods used by Zheng et al. [35] and Deng et al. [36] on different crops. This experiment found that the combination of a 15% reduction in irrigation amount, an OF application rate of 8 t·ha^−1^, and a CNF application rate of 306 kg·ha^−1^ performed best in terms of overall comprehensive performance, as determined by the CRITIC-AHP-VIKOR model, and achieved relatively high values in soil carbon and nitrogen pools, yield, and NFP. This indicates that in an integrated management of water and fertilizer under drip irrigation with plastic film mulching, appropriately reducing irrigation amount by 15% and NFP rate by 15% while applying OF, can achieve a synergistic effect between resource conservation and high yield with high efficiency, which is consistent with the conclusion of Zhang et al. [37] regarding nitrogen reduction and efficiency enhancement. However, excessive nitrogen reduction (e.g., N1) or excessive reduction in irrigation amount (e.g., W1) lead to a significant decrease in yield. This indicates that there is a threshold for the coupling effect among irrigation amount, nitrogen fertilizer, and organic fertilizer, and a balance needs to be sought between water conservation, nitrogen reduction, and yield assurance. The optimization model constructed in this study provides empirical evidence for the multi-objective decision-making advocated by Liao et al. [38], demonstrating that in the Hexi Oasis irrigation area, adopting the integrated technology of water and fertilizer under drip irrigation with plastic film mulching, moderate water-saving, nitrogen reduction, and OF substitution (irrigation amount of 4335 m^3^·ha^−1^, organic fertilizer application rate of 8 t·ha^−1^, and NFP rate of 306 kg·ha^−1^) is a sustainable strategy for balancing maize yield, soil carbon and nitrogen pools, grain quality, and resource efficiency.

Furthermore, it should be further noted that the comprehensive evaluation results in this study may be influenced by the selected indicator system and weighting method. Future studies could incorporate region-specific production objectives, expert preferences, and multiple weighting approaches to conduct sensitivity analyses and further verify the robustness of the recommended management scheme. Several limitations of this study should be acknowledged. First, the conclusions were derived from a two-year field experiment using a single maize cultivar in a typical oasis irrigation area of Zhangye. Therefore, the recommended water and fertilizer combination is primarily applicable to arid oasis agricultural regions with similar climatic conditions, soil properties, cropping systems, and irrigation practices. Its applicability to other maize cultivars, soil types, and agroecological regions requires further validation through long-term, multi-site, and multi-cultivar experiments. Second, interannual climatic variability may influence crop responses to irrigation reduction and organic fertilizer substitution, whereas the relatively short experimental period may not fully capture the long-term accumulation and stabilization of soil carbon and nitrogen. Third, this study mainly evaluated crop yield, grain quality, soil fertility, and resource use efficiency, without directly measuring greenhouse gas emissions, soil microbial community composition, enzyme activities, or carbon and nitrogen transformation processes. Future research should integrate these indicators to clarify the microbial and biogeochemical mechanisms underlying water–fertilizer interactions and to comprehensively assess the productivity, environmental sustainability, and long-term stability of the recommended management strategy.

## 4. Materials and Methods

### 4.1. Experimental Site Profile

**Figure 7:**
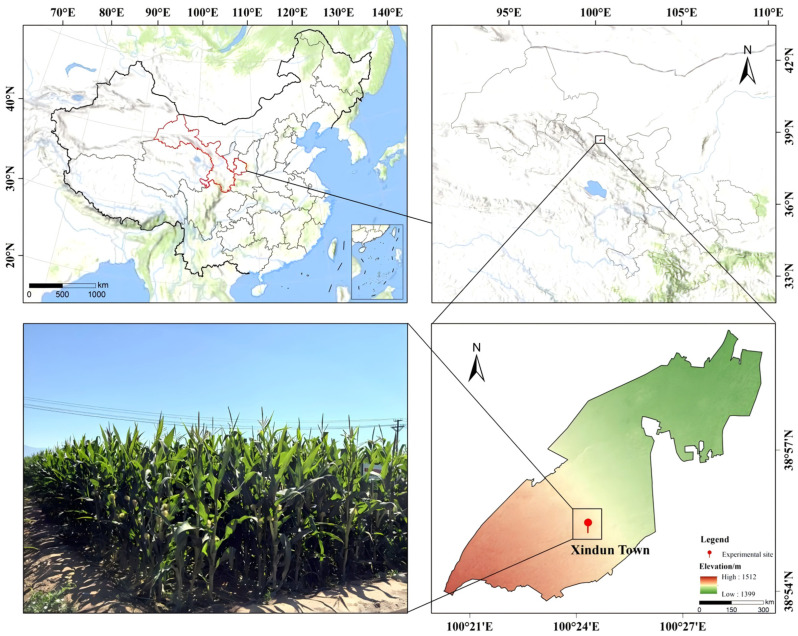
Location of the experimental site.

**Figure 8:**
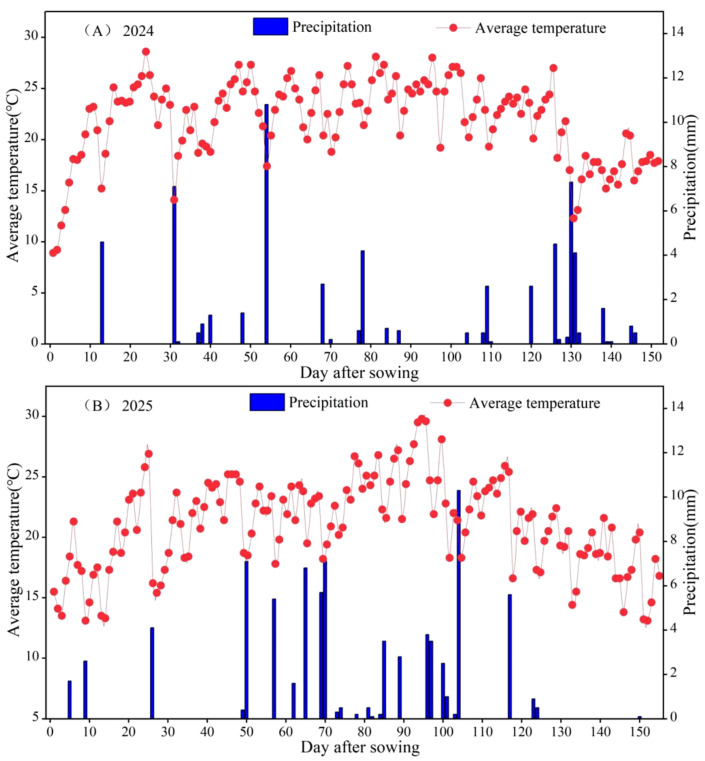
Daily variation in average temperature and precipitation throughout the maize growing seasons of 2024 (**A**) and 2025 (**B**).

The field experiment was carried out in Xindun Town, Zhangye City, Gansu Province (38°55′ N, 100°24′ E), a representative oasis agricultural area in Northwest China (Figure 7). The site is situated in the eastern Hexi Corridor at an elevation of approximately 1590 m and is characterized by a temperate continental arid climate. The region receives abundant solar radiation and heat resources, with a mean annual temperature of 7.2 °C, annual precipitation of approximately 120 mm, and annual evaporation of about 2200 mm. Rainfall is unevenly distributed throughout the year and exhibits pronounced seasonal variability. The soil at the experimental site is classified as fine sandy loam. Within the 0–20 cm soil layer, the pH and electrical conductivity were 8.47 and 2.03 mS·cm^−1^, respectively. Soil bulk density was 1.47 g·cm^−3^, and field capacity was approximately 22.89%. The basic soil nutrient comprises SOM of 9.2 g·kg^−1^, TN 0.89 g·kg^−1^, available phosphorus 30.1 mg·kg^−1^, alkali-hydrolyzable nitrogen 42.4 mg·kg^−1^, and available potassium 426.1 mg·kg^−1^. Cumulative precipitation during the maize growing season was 62.0 mm in 2024 and 79.0 mm in 2025. Seasonal variations in precipitation and mean temperature during the experimental period are shown in Figure 8.

### 4.2. Experimental Scheme

The maize variety tested was ‘Xianyu 1483’. The experiment adopted a randomized block design with two factors: reduced irrigation and organic fertilizer substitution for nitrogen fertilizers. Reduced irrigation was set up with three gradients: 30% irrigation amount reduction (W1: 3575 m^3^·ha^−1^), 15% irrigation amount reduction (W2: 4335 m^3^·ha^−1^), and conventional amount irrigation (W3: 5100 m^3^·ha^−1^). The substitution of OF for CNF was set up with three gradients: full application of CNF (N1: 360 kg·ha^−1^), 15% substitution with OF (N2: 306 kg·ha^−1^ of CNF + 8 t·ha^−1^ of OF), and 30% substitution with OF (N3: 252 kg·ha^−1^ of CNF + 16 t·ha^−1^ of OF). Each treatment was replicated three times, with a total of 27 plots, each plot covering an area of 24 m^2^ (6 m × 4 m). The water and fertilizer supply details for each treatment are shown in Table 3.

**Figure 9:**
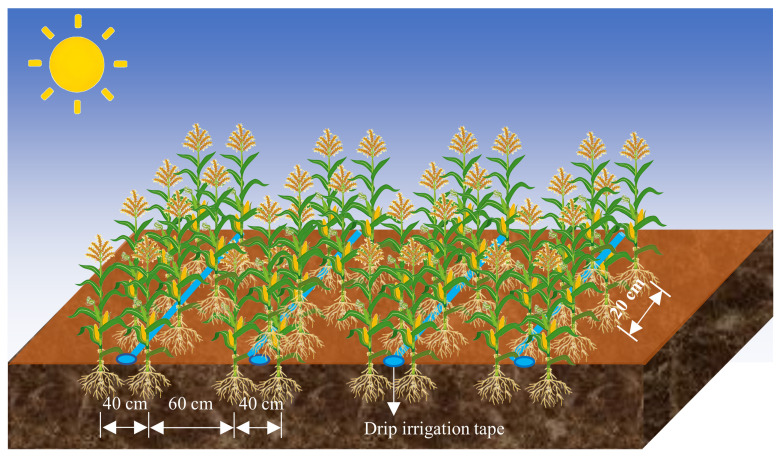
Schematic diagram of maize planting pattern.

Maize was sown on 28 April 2024, and 26 April 2025. Seedling emergence irrigation was applied on April 30. Harvest took place on 21 September 2024, and 26 September 2025. The planting method is in a wide and narrow row pattern. The spacing between the narrow rows is 40 cm, and the spacing between the wide rows is 60 cm. The plant spacing within each row is 20 cm, and the sowing density is 10.0 × 10^4^ plants·ha^−1^. Fertilization and irrigation were carried out using the integrated management of water and fertilizer under drip irrigation with plastic film mulching. The drip tape was of the embedded patch type, with a drip head spacing of 35 cm and a flow rate of 2.5 L·h^−1^ for each drip head. From June 10 to August 30, irrigation was applied once every 10 days, for a total of 8 irrigation events. Each irrigation amount was one-eighth of the designed total irrigation amount. The schematic diagram of the planting pattern is shown in Figure 9.

### 4.3. Experimental Materials

CNF applied during the experiment included urea (N ≥ 46%), superphosphate (P_2_O_5_ ≥ 12%), and potassium sulfate (K_2_O ≥ 24%). The application rates of phosphate and potassium fertilizers were the same for all treatments, at 180 kg·ha^−1^. All fertilizers were applied in their pure form and used as basal fertilizer. In addition, fully decomposed sheep manure was applied as a base fertilizer. Fully decomposed sheep manure was used as the OF for equal-N substitution, with a C/N ratio of 19.1:1, a moisture content of 23.18%, and a pH of 7.91. Before sowing, it was evenly spread on the surface of the experimental field and then incorporated into the soil at a depth of 35 cm using a rotary tiller. The basic characteristics of the OF were nitrogen ≥ 0.7% and organic matter ≥ 44%. The design of substituting CNF with OF in this study was based on the principle of equal nitrogen substitution calculated between fully decomposed OF and CNF.

### 4.4. Indicators Measurement and Methods

#### 4.4.1. Yield and Its Components

At the maturity stage, a sample plot of 6 square meters was set up in each designated area. Parameters assessed were ear length and kernel number per ear. After threshing and drying, the 100-kernel weight was measured (normalized to 14% moisture). The grain moisture content was analyzed using the PM8818 moisture meter (The instrument was a PM-8188NEW model manufactured by Shanghai Jiashi Electronic Technology Co., Ltd., Shanghai, China), and yield was subsequently calculated.

#### 4.4.2. Soil Organic Matter and Total Nitrogen

At the maturity stage, soil core samples were collected from the 0–60 cm profile using a soil auger and divided into three depth layers (0–20, 20–40, and 40–60 cm). After naturally air-dried, the samples were passed through a 0.15 mm sieve. SOM content was determined using the potassium dichromate oxidation method, and TN was quantitatively analyzed using the semi-micro Kjeldahl nitrogen determination method [39].

#### 4.4.3. Quality

At the maturity stage, samples were collected, dried, and finely ground. The crude fat content was analyzed using the Soxhlet extraction method, the crude protein content was determined by the Kjeldahl nitrogen determination method, and the crude starch contents were quantitatively determined using the anthrone-sulfuric acid colorimetric method [40,41].

#### 4.4.4. Water and Nitrogen Use Efficiency

(1) Irrigation water use efficiency (*IUE*, kg·m^−3^),
  (1) IUE=Y/W
  where *W* is the irrigation volume, m^3^; *Y* is the yield, kg·ha^−1^.

(2) Nitrogen fertilizer partial productivity (NFP, kg·kg^−1^),
  (2) NFP=Y/N
  where *N* is the nitrogen fertilizer input, including the TN content of both chemical and organic fertilizers, kg·ha^−1^.

### 4.5. Multi-Objective Comprehensive Evaluation Method

#### 4.5.1. CRITIC Weighting Method

(1) Data standardization [42]

① Positive indicators:

(3) $$r_{ij}^{*}=\frac{r_{ij}-\min j}{\max j-\min j}$$

② Negative indicators:

(4) $$r_{ij}^{*}=\frac{\max j-r_{ij}}{\max j-\min j}$$

(2) Calculate the variability of the indicators (standard deviation *S_j_*):
  (5) $$S_{j}=\sqrt{\frac{\sum_{i=1}^{n}{\left(x_{ij}-{\bar{x}}_{j}\right)}^{2}}{n-1}}$$
  where *i* represents the number of treatments; $x_{ij}$ is the *j*-th indicator of the *i*-th treatment; ${\bar{x}}_{j}$ is the mean value of the indicators.

(3) Calculate the conflict index (*R_j_*):
  (6) $$R_{j}=\sum_{i=1}^{n}\left(1-r_{ij}\right)$$
  where $r_{ij}$ represents the correlation coefficient between evaluation indicators *i* and *j*.

(4) Information entropy (*C_j_*):
  (7) $$C_{j}=S_{j}\sum_{i=1}^{n}\left(1-r_{ij}\right)=S_{j}\times R_{j}$$

(5) Weight (*W_j_*):
  (8) $$W_{j}=\frac{C_{j}}{\sum_{j=1}^{n}C_{j}}$$

#### 4.5.2. AHP Comprehensive Evaluation Method

(1) Construction of the judgment matrix

Using the 1–9 scoring method (Table 4), five experts in maize cultivation and soil fertilization were invited to conduct pairwise comparisons of the importance of 11 evaluation indicators, and a 11th-order reciprocal judgment matrix A = (aij) n × n was constructed (Table 5). Here, *n* = 11 represents the total number of indicators, and aij represents the importance of the i-th indicator relative to the j-th indicator. This judgment matrix satisfies the following properties [42].

(9) $$a_{ii}=1$$

(10) $$a_{ji}=\frac{1}{a_{ij}}\;\;\;a_{ij}>0\;(i,j=1,2,\ldots ,n)$$

(2) Normalization of column vectors

(11) $${\bar{a}}_{ij}=\frac{a_{ij}}{\sum_{k=1}^{n}a_{kj}}\;(i,j=1,2,\ldots ,n)$$

(3) Perform summation and normalization (weights)

(12) $$w_{i}=\frac{\sum_{j=1}^{n}{\bar{a}}_{ij}}{\sum_{k-=1}^{n}\sum_{j=1}^{n}{\bar{a}}_{ij}}\;(i=1,2,\ldots ,n)$$

(4) Calculate the maximum eigenvalue
  (13) $$\lambda_{\max}=\frac{1}{n}\sum_{i=1}^{n}\frac{{\left(AW\right)}_{i}}{w_{i}}$$
  where ${\left(AW\right)}_{i}$ represents the *i*-th component of the product of the judgment matrix *A* and the weight vector *W*.

(5) Consistency Check
  (14) $$CI=\frac{\lambda_{\max}-n}{n-1}$$
  (15) $$CR=\frac{CI}{RI}$$
  where *CI* represents the consistency index, and, in this study, the calculated value of *CI* is 0.142; *RI* is the average random consistency index, and, for this study, *n* = 11, *RI* = 1.51; *CR* is the consistency ratio. The final calculation yields *CR* = 0.094 < 0.1, passing the one-time test.

#### 4.5.3. VIKOR Comprehensive Evaluation Method

(1) Data standardization

Data standardization followed Equations (3) and (4) as described above.

(2) Compute the group utility measure (*S*) and the individual regret measure (*R*) [43,44]
  ① Positive indicators:
  (16) $$S_{i}=\sum_{j=1}^{n}W_{j}\cdot \frac{f_{j}^{+}-r_{ij}}{f_{j}^{+}-f_{j}^{-}}$$
  (17) $$R_{i}=\max j\left(W_{j}\cdot \frac{f_{j}^{+}-r_{ij}}{f_{j}^{+}-f_{j}^{-}}\right)$$
  ② Negative indicators:
  (18) $$S_{i}=\sum_{j=1}^{n}W_{j}\cdot \frac{r_{ij}-f_{j}^{+}}{f_{j}^{+}-f_{j}^{-}}$$
  (19) $$R_{i}=\max j\left(W_{j}\cdot \frac{r_{ij}-f_{j}^{+}}{f_{j}^{+}-f_{j}^{-}}\right)$$
  where $f_{j}^{+}$ and $f_{j}^{-}$ represent the positive and negative ideal solutions, respectively, for the *j*-th evaluation index.
(3) Calculate the profit ratio (*Q*):
  (20) $$Q_{i}=\upsilon \cdot \frac{S_{i}-S_{\min}}{S_{\max}-S_{\min}}+(1-\upsilon )\cdot \frac{R_{i}-R_{\min}}{R_{\max}-R_{\min}}$$
  where *v* represents the decision-making mechanism factor, *v* = 0.5.

### 4.6. Data Analysis

In this study, we conducted data collection and analysis in at least three independent replicates. Preliminary data management and analysis were performed using Microsoft Excel 2016 and IBM SPSS Statistics 26, respectively. A two-factor analysis of variance (ANOVA) was employed, with a significance level of *p* < 0.05, and Duncan’s multiple range test was conducted. Graphs and charts were created using Origin 2021, ArcMap 10.3, and Microsoft PowerPoint 2016 software.

## 5. Conclusions

This study identified the medium irrigation combined with medium organic fertilizer substitution scheme (W2N2) as a promising water- and nitrogen-saving strategy for maize production in the arid regions of Northwest China. This scheme not only maintained high levels of soil carbon and nitrogen pool indicators, such as SOM and TN, but also significantly increased yield by 3.55–54.38%, reaching a maximum of 19.56 t·ha^−1^. The study also indicated that the application of OF is a key factor influencing the effectiveness of nitrogen fertilizer. Under the condition of moderate application of OF, appropriately reducing the amount of CNF and irrigation can maximize the positive effects of nitrogen resource use. However, when the amount of OF is insufficient, excessive irrigation will lead to a decrease in yield and efficiency. The correlation analysis showed that maize yield was significantly positively correlated with its components, SOM, TN, grain crude protein content, crude starch content, crude fat content, IUE, and NFP, although the correlation between IUE and other indicators did not reach a significant level. The CRITIC-AHP-VIKOR multi-objective comprehensive evaluation model confirmed that the W2N2 treatment achieved the best overall performance. Therefore, in maize production in the Hexi Corridor irrigation area, it is recommended to adopt an optimal water and fertilizer management model with a 15% reduction in irrigation amount (4335 m^3^·ha^−1^) and a 15% substitution rate of chemical fertilizer with organic manure (organic manure: 8 ·ha^−1^; chemical fertilizer nitrogen: 306 kg·ha^−1^). This treatment maintained high yield while improving soil carbon and nitrogen pools, grain quality, and resource use efficiency, thereby supporting sustainable agriculture.

## Figures and Tables

**Table 1:** Effects of reduced irrigation and OF substitution for CNF on maize yield and its components.

Treatment	2024				2025			
	Ear Length (cm)	Kernel Number Per Ear	100-Kernel Weight (g)	Yield (t·ha^−1^)	Ear Length (cm)	Kernel Number Per Ear	100-Kernel Weight (g)	Yield (t·ha^−1^)
W1N1	14.74 ± 0.40 c	442 ± 14.36 b	32.60 ± 1.47 b	13.25 ± 0.40 b	14.29 ± 0.63 c	448 ± 16.26 c	31.47 ± 0.58 e	12.08 ± 0.13 d
W1N2	16.20 ± 0.49 abc	460 ± 5.00 b	35.80 ± 1.26 b	14.16 ± 0.38 b	15.93 ± 0.53 abc	476 ± 7.75 bc	34.56 ± 0.86 d	13.27 ± 0.27 c
W1N3	15.92 ± 0.59 bc	452 ± 14.17 b	34.18 ± 0.91 b	14.02 ± 0.45 b	15.60 ± 0.48 bc	486 ± 13.04 abc	34.09 ± 0.61 de	13.03 ± 0.28 cd
W2N1	16.16 ± 0.53 abc	508 ± 17.16 a	36.23 ± 0.97 b	18.97 ± 0.76 a	16.42 ± 0.44 ab	504 ± 15.50 ab	35.95 ± 0.22 bcd	17.85 ± 0.19 b
W2N2	17.84 ± 0.49 a	526 ± 21.99 a	42.10 ± 1.48 a	19.39 ± 0.30 a	17.51 ± 0.36 a	530 ± 7.80 a	39.30 ± 1.59 a	19.72 ± 0.51 a
W2N3	17.26 ± 0.58 ab	516 ± 11.93 a	41.83 ± 0.66 a	18.06 ± 0.28 a	17.34 ± 0.71 ab	522 ± 20.69 ab	38.46 ± 0.93 abc	18.86 ± 0.67 ab
W3N1	15.85 ± 0.53 bc	502 ± 12.50 a	35.38 ± 0.79 b	18.63 ± 0.44 a	16.11 ± 0.48 ab	512 ± 6.25 ab	35.72 ± 0.73 cd	18.59 ± 0.45 ab
W3N2	17.49 ± 0.52 ab	520 ± 14.24 a	41.95 ± 0.81 a	18.74 ± 0.53 a	17.29 ± 0.48 ab	518 ± 21.17 ab	38.80 ± 0.77 ab	19.03 ± 0.31 ab
W3N3	17.13 ± 0.64 ab	508 ± 9.24 a	40.80 ± 1.62 a	18.15 ± 0.48 a	17.08 ± 0.60 ab	504 ± 9.29 ab	35.83 ± 1.36 bcd	18.72 ± 0.11 ab
Significance (F)								
W	**	***	***	***	**	**	***	***
N	**	ns	***	ns	*	ns	**	**
W × N	ns	ns	ns	ns	ns	ns	ns	ns

Note: Lowercase letters in the table indicate significant differences among different treatments. W represents the irrigation amount. N represents the rate of OF substituting CNF. W × N represents the interaction effect between the irrigation amount and the rate of OF substituting CNF. * indicates significance at the *p* < 0.05 level. ** indicates high significance at the *p* < 0.01 level. *** indicates a highly significant level at the *p* < 0.001 threshold. ns indicates no significance at the *p* > 0.05 level. The same applies below.

**Table 2:** Comprehensive evaluation based on CRITIC-AHP-VIKOR model.

Treatment	S	R	Q	Sort
W1N1	1.000	0.164	1.000	9
W1N2	0.632	0.139	0.729	7
W1N3	0.671	0.143	0.765	8
W2N1	0.365	0.077	0.387	3
W2N2	0.015	0.015	0.000	1
W2N3	0.148	0.032	0.126	2
W3N1	0.504	0.109	0.563	6
W3N2	0.226	0.102	0.399	4
W3N3	0.323	0.113	0.486	5

Note: *S* represents the group utility value; *R* represents the individual regret value; *Q* represents the benefit ratio value. The comprehensive evaluation of each treatment is based on the average of data from 2024 and 2025.

**Table 3:** The experimental scheme.

Treatment	Irrigation Mount (m^3^·ha^−1^)		Nitrogen Application Rate	
	2024	2025	CNF (kg·ha^−1^)	OF Addition (t·ha^−1^)
W1N1	3575 (Reduce irrigation amount by 30%)	3728 (Reduce irrigation amount by 30%)	360	0 (Apply CNF throughout)
W1N2			306	8.0 (OF replaced 15% CNF)
W1N3			252	16.0 (OF replaced 30% CNF)
W2N1	4335 (Reduce irrigation amount by 15%)	4526 (Reduce irrigation amount by 15%)	360	0
W2N2			306	8.0
W2N3			252	16.0
W3N1	5100 (Regular irrigation)	5325 (Regular irrigation)	360	0
W3N2			306	8.0
W3N3			252	16.0

Note: Due to the difference in rainfall between the two growing seasons, in order to maintain the rigor of the experiment, the irrigation water volumes for 2024 and 2025 were adjusted simultaneously to ensure that the amount of water that seeps into the soil layer in the two years was the same. Meanwhile, the figures used in the article for the first year’s irrigation water volume represent the theoretical value of the annual irrigation requirement.

**Table 4:** The meanings of the AHP 1–9 scale method.

Scale	Meaning
1	Both indicators are equally important
3	When comparing the two indicators, the former is slightly more important
5	The former indicator is significantly more important than the latter
7	The former indicator is strongly more important than the latter
9	The former indicator is extremely more important than the latter
2, 4, 6, 8	Intermediate values between the above adjacent judgments
Inverted	If indicator *i* is compared with indicator *j* and the result is a*_ij_*, then the comparison of *j* with *i* is a*_ji_* = 1/a*_ij_*

**Table 5:** The index matrix of each indicator in the AHP method.

Indicators	EI	SGN	WHG	Y	SOM	TN	P	S	CF	IUE	NFP
EI	1	0.5	0.5	0.25	2	2	2	1	1	1	1
SGN	2	1	1	2	3	3	1	2	2	2	2
WHG	2	1	1	2	3	4	1	2	2	2	2
Y	4	0.5	0.5	1	6	8	2	5	4	4	0.25
SOM	0.5	0.333	0.333	0.167	1	1	3	1	2	1	1
TN	0.5	0.333	0.25	0.125	1	1	4	2	2	0.5	0.5
P	0.5	1	1	0.5	0.333	0.25	1	2	2	2	0.5
S	1	0.5	0.5	0.2	1	0.5	0.5	1	1	1	1
CF	1	0.5	0.5	0.25	0.5	0.5	0.5	1	1	1	1
IUE	1	0.5	0.5	0.25	1	2	0.5	1	1	1	1
NFP	1	0.5	0.5	0.25	1	2	0.5	1	1	1	1

## Data Availability

The original contributions presented in the study are included in the article, further inquiries can be directed to the corresponding author.
